# Clinical Profile of Tuberculum Sellae Meningiomas Based on Scoring System: An Institutional Experience in Indonesia

**DOI:** 10.3390/cancers15235700

**Published:** 2023-12-03

**Authors:** Renindra Ananda Aman, Risayogi W. A. H. Sitorus, Muhamad Aulia Rahman, Fabianto Santoso, Ramadhan Kurniawan

**Affiliations:** Department of Neurosurgery, Faculty of Medicine, Universitas Indonesia—Dr. Cipto Mangunkusumo National General Hospital, Jakarta 10430, Indonesia; risayogi.sitorus@gmail.com (R.W.A.H.S.); auliarahmanmd@gmail.com (M.A.R.); fabianto.santoso@ui.ac.id (F.S.); ramadhankunni@gmail.com (R.K.)

**Keywords:** tuberculum sellae meningioma, scoring system, invasion, prognosis

## Abstract

**Simple Summary:**

Tuberculum sellae meningioma is a condition that typically results in visual impairment as well as other symptoms, such as hemianopsia and parasellar extension. This study aimed to show the epidemiology and clinical profile of tuberculum sella meningioma at Indonesia’s National Referral Hospital. This study also used the TSM scoring system to describe tumor severity and predict outcomes. Canal invasion is associated with visual impairment symptoms, and both canal and vascular invasion can impact the resectability of the tumor. Other factors that may influence the patient’s visual outcome include the onset of visual symptoms, peritumoral edema, and grade of excision. Hence, this study can be utilized to develop a scoring system to predict the severity and outcomes of patients after surgery.

**Abstract:**

Tuberculum sellae meningioma (TSM) is a challenging tumor that grows close to several crucial structures, such as the optic nerve, arteries, and pituitary. Surgical treatment is currently evolving from a transcranial microsurgical resection to a transsphenoidal approach. This study examined the clinical profile of patients with tuberculum sellae meningioma and explored its relationship with scoring systems. This retrospective observational study included patients with TSM who underwent surgery at the Department of Neurosurgery at our hospital between 2017 and 2022. The patients were excluded if their data required completion. The clinical profiles of the patients were counted and transformed into a scoring system using several variables such as size, vascular, and canal invasion. We then analyzed the relationship between the clinical signs and symptoms to determine the efficacy of this scoring system. Thirty-six patients were included in the study. Most of our patients had a high score for tumor diameter, bilateral canal invasion, and vascular invasion (2-2-2). Moreover, when related to clinical signs, there was no relationship between the canal and vascular invasion and decreased visual acuity. Tuberculum sellae meningioma mostly causes visual impairment and several other symptoms, such as hemianopsia and parasellar extension. Several factors in the scoring system should also be considered to predict outcomes, such as the onset of visual symptoms, peritumoral edema, and grade of excision.

## 1. Introduction

Tuberculum sellae meningioma (TSM) accounts for 5–10% of all intracranial meningiomas. Meningioma is a slow-growing tumor of around 2–4 mm/year for asymptomatic meningioma [1]. It arises from the tuberculum sellae, sulcus chiasma, limbus sphenoidal, and diaphragm sellae and grows in a sub-chiasmal position [2,3]. Tuberculum sellae meningioma (TSM) is a challenging tumor because of its proximity to the optic nerve, internal carotid artery, and anterior cerebral artery, as well as to other structures such as the hypothalamus, infundibulum, and pituitary gland [4]. Small tumors can also press on the optic nerve. Visual disturbances are the main indication for surgical management. Surgical techniques for TSM are evolving from transcranial microsurgical resection to an endoscope through a transsphenoidal approach [2,3,5]. Transcranial surgery can be performed through bifrontal, interhemispheric, orbitozygomatic, pterional, and sub-frontal eyebrow approaches. With the advancement of endoscopy and the development of extended endonasal approaches, there is still a debate as to the best surgical management via a transcranial or endonasal and/or transsphenoidal approach. However, the specific considerations and indications for the patient and the description of the tumor undergoing transcranial or transsphenoidal surgery were very individualized and currently remain unclear, with no definite guidelines. Radiosurgery, such as gamma knife, could be a feasible option in treating patients with high difficulty for traditional surgery [5,6]. Several parameters that can be considered for the scoring system include tumor size, invasion of the optic canal, vascular invasion, and peritumoral edema [7]. This study aimed to determine the clinical profile and characteristics of tuberculum sellae meningioma in Dr. Cipto Mangunkusumo National General Hospital and their relation to the scoring system.

## 2. Materials and Methods

**Figure 1:**
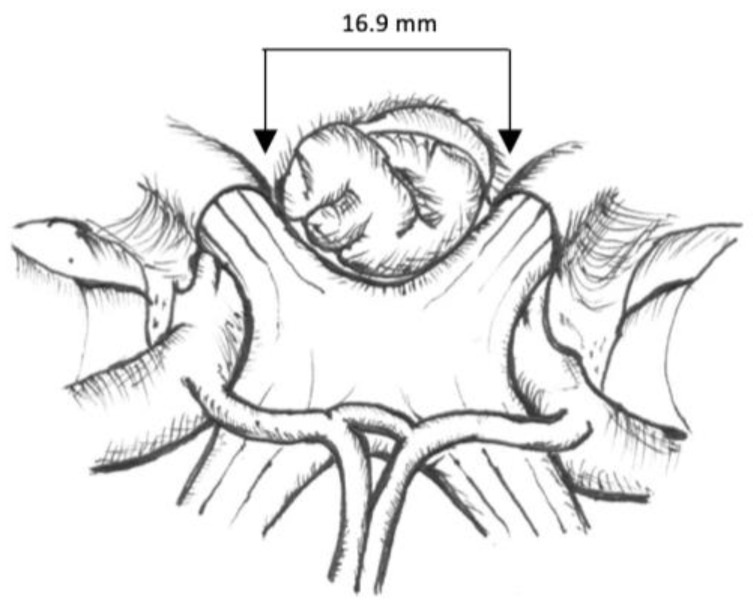
Proposed TSM scoring score: Tumors < 16.9 mm in diameter received 1 point. Tumors ≥ 16.9 mm in diameter received 2 points. The mean intercanalis optic distance was 16.9 mm (range 15.94–17.83 mm).

**Figure 2:**
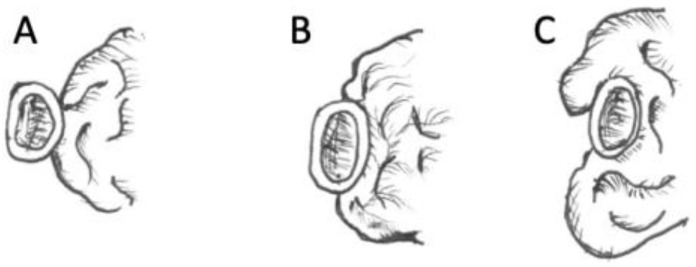
Proposed TSM scoring score: artery score (ICA and ACA); tumors receive 0 points when abuts the medial wall only (**A**); 1 point when envelopes < 180° around artery (**B**), and 2 points when encasing ≥ 180° around artery (**C**) [8].

This retrospective study included all patients with tuberculum sellae meningioma who underwent transcranial surgery between 2017 and 2022 at our department at Dr. Cipto Mangunkusumo National General Hospital, Jakarta, Indonesia. Patients were excluded if their medical records, neuro-ophthalmology examinations, or radiological data were incomplete. This research was approved by the Faculty of Medicine Universitas Indonesia ethics committee in protocol number 22-07-0707. We analyzed the clinical profile, tumor size, and scoring classification, including the tumor size, canal invasion, and vascular invasion. Peritumoral edema was also assessed. Data were analyzed using the Chi-square or Fisher analysis for categorical variables and the unpaired *T*-Test or Mann–Whitney test for numeric data. The scoring system used was that described by Magill et al. (Figure 1 and Figure 2, Table 1).

## 3. Results

There were 36 tuberculum sella meningiomas; however, only 29 patients were included for additional analysis due to the availability of complete data, with a mean of age 45.1 ± 7.4 years old (Table S1). Preoperative tumor size was 17.18 cc (range 2.4–68 cc), and postoperative tumor size was 1.03 cc (range 0–65 cc), with a reduction rate of 91%. Most of the patients were female (91.7%), and 88.9% had a reduction in visual acuity (<3/60). Hemianopsia was found in 50% of patients; however, 25% could not be examined owing to the lack of light perception of visual acuity. Parasellar extension was observed in only 5.6% of the patients (Table 2). In our study, we calculated the internal optic distance in all patients, with a mean of 16.9 mm (range 15.94–17.83 mm). We then applied the tuberculum sellae meningioma scoring system according to Magill et al., based on tumor size, canal, and vascular invasion, as shown in Table 3. Peritumoral edema was observed in only 6.9% of the patients.

We also analyzed the relationship between the scoring system and clinical signs such as visual acuity and hemianopsia. There was no significant relationship between optic canal invasion, decreased visual acuity (*p* = 0.076), and vascular invasion (*p* = 0.622) (Table 4). The tumor diameter showed no relationship with any clinical signs. The onset of symptoms had no significant relationship with visual acuity or hemianopsia; however, patients with a more significant onset had higher scores (Table 5). For vascular invasion, the higher score group had a longer onset duration and a lower resection rate (Table 6). The canal invasion score was associated with the resection rate (*p* < 0.001). None of the scoring items demonstrated a correlation with clinical improvement (Table 7).

In this study, all histopathological profiles were determined to be meningioma WHO grade 1, with the majority being of the meningothelial type (51.7%) (Table 8). The remaining profiles were a mixed type of meningothelial, microcystic, transitional, and fibrous meningioma, all of which were classified as WHO grade 1.

## 4. Discussion

Tuberculum sellae meningiomas are challenging tumors to resect, but they can be removed with acceptable morbidity and mortality, representing marked progress from the historical series [2]. The tuberculum sellae is bounded laterally by the clinoid processes, internal carotid, and posterior communicating arteries with the arachnoid of the carotid cisterns, posteriorly by the pituitary stalk, infundibulum, and Liliequist membrane and superiorly by the optic chiasm, lamina terminalis, and ACA complex. This space was relatively small (mean length, 8 mm; mean width, 11 mm). The path of least resistance for tumor growth tends to be over the planum sphenoidal anteriorly (perhaps due to a defect in the chiasmatic cistern arachnoid), around the optic nerves, sometimes into the optic canals laterally, above the chiasm, displacing it superiorly, and down over the tuberculum and seller inferiorly [4,8,9,10].

The most common symptoms of TSM are visual disturbances such as decreased visual acuity and visual field defects. There is a broad pattern of visual deterioration, in which vision loss occurs gradually in one eye, followed gradually in the contralateral eye. The patients could also experience headaches; neurological deficits; altered behavior; or sensorium, anosmia, seizures, and endocrine disturbances [3,11,12].

Meningiomas are commonly classified according to the World Health Organization (WHO) classification of CNS tumors. The grading system was fundamentally based on the malignancy level of the tumor, which marked the important role of pathological anatomy in the complete diagnosis of TSM, even though the analysis was more commonly performed during or after the resection of the neoplasm. Meningioma was classified into three grades: grade 1 for benign types, grade 2 for atypical types, and grade 3 for malignant or anaplastic types. Grade 1 is comprised of meningothelial, fibrous, transitional, psammomatous, angiomatous, microcystic, secretory, lymphoplasmacyte-rich, and metaplastic meningiomas; grade 2 is comprised of chordoid, clear cell, and atypical meningiomas; and grade 3 is comprised of papillary, rhabdoid, and anaplastic meningiomas [13]. In this study, all patients were diagnosed with meningioma WHO grade 1, with the majority classified as meningothelial meningiomas.

The tumor scoring score for tuberculum sellae meningioma is currently used to determine the best approach for tumor removal. Magill et al. showed that a higher score was found in the transcranial group, while a lower score was found in the transsphenoidal group [9,14,15]. Postoperative visual function is the most important surgical outcome following the resection of TSM [16]. Therefore, to create a better outcome, we should evaluate the invasion of the optic canal [8]. This scoring system was created based on the authors’ experience in resecting TSMs and the anatomical literature. The scoring is simple and combines three key tumor characteristics that determine the difficulty of re-section: tumor size, optic canal invasion, and arterial encasement. The tumor size score was 1 or 2 points: 1 point if the tumor was <16.9 mm in diameter and 2 points if it was ≥16.9 mm. The diameter of 16.9 mm was selected based on two anatomical studies that measured the average distance between the optic nerves at the limbus sphenoidal; in our study, the mean intercanal optic distance was found to be 16.9 (range 15.94–17.83 mm) [8,16]. The optic canal invasion score is graded as follows: if the tumor invades ≤ 3 mm into either optic canal, then it receives 0 points; if it invades > 3 mm into one optic canal, it receives 1 point; and if it invades both optic canals > 3 mm, it receives 2 points [8]. The intercanalis optic nerve distance in our study was 16.9 mm, which can be applied to this scoring system to predict the approach and outcome of surgery [8]. However, we did not use this scoring system to determine the decision of our surgical approach. Instead, it was solely used for the classification and clinical profile of our patients. All surgeries were performed using the transcranial approach.

In this study, we found no significant relationship between higher scores for optic canal invasion and decreased visual acuity or vascular invasion (Table 3). Several studies have described various factors affecting visual outcomes, such as young age and short symptom duration, which are good prognostic factors for visual outcomes [2,16,17,18]. In addition, preoperative visual function, anterior cerebral artery encasement, and tumor size must be considered as influencing factors. Several studies have shown that patients with symptoms for more than seven months showed unfavorable outcomes [19]. Our study did not find a statistically significant relationship between the onset duration and visual impairment. However, higher scores for canal invasion, vascular invasion, and tumor size were associated with a longer duration of onset (Table 4). A possible reason for this is that compressive mechanical injury leads to small vessel compromise and demyelination, especially in patients with prolonged visual loss before surgery. Peritumoral edema in meningiomas is correlated with a higher proliferative index and angiogenic activity. Therefore, the dismal visual outcome may be related to a higher growth rate and accelerated course of nerve compression [2,8,19]. Magill ST (2018) reported that the larger the tumor, the higher the risk for visual worsening [8]. Chi et al. (2006) stated that the extension of the tumor into the optic canal correlated with significant visual defects [4]. However, this result might be due to the small sample size.

TSM mostly causes visual acuity impairment, and further extension pushing the optic chiasm can cause hemianopsia. In contrast, extension to the parasella can damage the nerve in the superior orbitalis fissure [8,20]. In our study, the tumor reduction rate was as high as 91%. In this study, gross total resection was achieved in the absence of vascular invasion (100%), as has been reported in other studies. A higher vascular invasion score was significantly associated with a lower resection rate than the other scores (*p* = 0.024). In contrast, the canal invasion score was significantly associated with the resection rate (*p* < 0.001). The same result was obtained in the study by Magill et al., who reported that a higher canal score decreased the likelihood of achieving gross total resection [8]. According to Palani et al. (2012) reported that the significant factors that influenced complete resectability include tumor size < 6 cm, peritumoral edema, arterial encasement, and surgical approaches—extended bifrontal and unilateral frontal. Other factors, such as brain–tumor interface, hyperostosis, and optic canal extension, do not significantly influence the complete resection of the tumor [21,22].

Our analysis of scoring items associated with clinical improvement revealed no statistically significant relationships. However, a larger tumor diameter, optic canal invasion, and vascular invasion were not found to be correlated with improved patient outcomes. This could be attributed to the limited sample size of our study and the delay in hospital admission, as our patients were typically admitted after experiencing severe symptoms, such as blindness. Consequently, the resection rate or success of surgery does not improve the symptoms.

As in many other intracranial tumors, surgical resection of the tumor is the definitive treatment for TSM. Microsurgical approaches encompassed the conventional transcranial approach and the more advanced extended endoscopic endonasal approach. The transcranial approach can be performed using subfrontal, bifrontal, frontotemporal, pterional, supraorbital, interhemispheric, orbitozygomatic, and mixed approaches. Both approaches have advantages and disadvantages. The transcranial approach is a classic and widely used approach, as it only requires standard neurosurgical tools. Using this technique, the neurosurgeon had more freedom of visual field and movement control compared to the endoscopic technique, especially in tumors on the optic apparatus or around the anterior clinoid process. Another strong advantage of transcranial surgery is the ability to reach a tumor that extends laterally beyond the optic nerves and internal carotid arteries, which cannot be reached via endoscopy. Brain edema was also a determining factor in choosing the surgical approach because disturbance of the arachnoid plane was a contraindication for the extended endoscopic endonasal approach. However, the use of extended endoscopic endonasal and/or transsphenoidal approaches has significantly increased in recent years. The endoscopic procedure provides the most direct field of view of the tumor site in the tuberculum sellae, which resulted in earlier tumor devascularization as the capsule could be dissected, and the tumor was debulked through the extraarachnoid route. Its minimal interference with healthy brain tissue and neurovascular structures made the procedure safer and resulted in less blood loss. The use of endoscopy was more sensitive in detecting post-resection tumor remnants and provided patients with cosmetic satisfaction. However, the anterior skull base and the underlying duramater had to be removed endoscopically. In many cases, the removal resulted in a higher risk of cerebrospinal fluid leakage or even the formation of fistula [20,23].

The tumor scoring system for tuberculum sellae meningioma, which was explained in a previous paragraph, was used to determine whether the patient is more suited to having transcranial or endoscopic surgery [8]. The first approach is more suitable if the patient has a large tumor above 4 cm, more than half vascular encasement, severe optic canal involvement, or brain invasion, while the latter approach is more suitable for patients with milder tumors [23]. For this reason, the transcranial approach was still considered the gold standard in TSM treatment [12].

In reality, comparing the outcomes of surgeries in different studies or even cases is often difficult to implement, considering the lack of uniformity of the disease and management in different individuals. The extended endoscopic endonasal approach has proven superior in many case reports and retrospective studies, although without statistical significance compared to traditional surgery. A study by Qian et al. showed statistical insignificance (*p* > 0.05) in gross total tumor resection, with 85.9% achieved with transcranial surgery and 91.2% with extended endoscopic endonasal surgery. A retrospective study also reported stable or improved visual acuity in 74 of 92 patients with TSM. Of these, 47 underwent transcranial surgery, and 27 underwent extended endoscopic endonasal surgery. The cases resulted in a ratio between patients with visual stabilization or improvement and those with visual worsening, which accounted for 47:16 in transcranial surgery and 27:2 in extended endoscopic endonasal surgery. The ratio showed a clear advantage with a good statistical significance (*p* = 0.038) [20,24].

Complete resection should not be attempted at the expense of visual deterioration or hypothalamic dysfunction. The percentage of the gross total tumor resection of these meningiomas ranged from 35% to 100% [25,26]. A retrospective study conducted in 2002 by Goel et al. showed an 84.3% rate of total resection [11] with recent advancements in technology. In a retrospective study conducted 20 years later by Qian et al., it was found that the traditional transcranial approach achieved a gross total resection rate of 85.9%, whereas the more recent extended endoscopic endonasal approach yielded a rate of 91.2%. However, there are conflicting reports regarding the impact of tumor size on visual outcomes [23]. Several authors have shown that larger tumors had worse outcomes while others were contrariwise [4,8,19,27,28]. Earlier surgical intervention was believed to provide better outcomes, especially in visual improvement. There were a few reported complications of resection surgery of TSM. In a 40-patient retrospective study of post-endoscopic surgery TSM patients, Yu et al. found hyposmia (20%) to be the most common complication, followed by CSF leak (7.5%, in which two-thirds of them developed meningitis), transient visual impairment (2.5%), and acute cerebral infarction (2.5%). They found no seizure or hypopituitarism [29]. Kenawy et al. analyzed the data of 17 post-transcranial surgery TSM patients and found 1 hematoma, 1 transient diabetes insipidus, and 1 status epilepticus resulting in death; however [12]. The mortality rate of tuberculum sellae meningioma was even more limited, but it was estimated at 0 to 67%, with a lower mortality rate achieved in recent times. TSM could also reoccur, with a recurrence rate of 0–25% [24].

Stereotactic radiosurgery, such as the gamma knife procedure or external beam therapy, could be another treatment option. Stereotactic radiosurgery is usually appropriate for patients with small to medium anterior skull base meningiomas. The procedure was performed to control the growth of the tumor and given in a few episodes over a period of time to allow for normal tissue regeneration between the doses [5,6,30,31].

A limitation of this study was the small sample size. Additional samples are required for further studies. Another limitation is that the standard approach for tuberculum sellae meningioma at our institution is the transcranial approach. None of the patients underwent the transsphenoidal approach. Therefore, we were unable to compare these approaches by using a scoring system.

## 5. Conclusions

Tuberculum sellae meningioma is a condition that typically results in visual impairment as well as other symptoms, such as hemianopsia and parasellar extension. Canal invasion is associated with visual impairment symptoms, and both canal and vascular invasion can impact the resectability of the tumor. Other factors that may influence the patient’s visual outcome include the onset of visual symptoms, peritumoral edema, and grade of excision.

## Figures and Tables

**Table 1:** The scoring system for tuberculum sellae meningioma [8].

**Tumor Score**	
1	Less than 16.9 mm
2	Greater/equal 16.9 mm
**Canal Score**	
0	No invasion or (≤3 mm)
1	Unilateral invasion > 3 mm
2	Bilateral invasion > 3 mm
**Artery Score**	
0	It abuts the medial wall only
1	Envelops < 180° around the artery
2	Encases ≥ 180° around the artery

**Table 2:** Clinical profiles of tuberculum sellae patients in Dr. Cipto Mangunkusumo National General Hospital, 2017–2022.

Variable	Number (%)
**Gender**	
Male	3 (8.3)
Female	33 (91.7)
**Visual acuity**	
≥3/60	4 (11.1)
<3/60	32 (88.9)
**Hemianopsia**	
Yes	18 (50)
No	9 (25)
N/A	9 (25)
**Parasellar Extension**	
Yes	2 (5.6)
No	34 (94.4)

**Table 3:** Application to tuberculum Mmeningioma scoring system.

Scoring	Number (%)
**Tumor Diameter**	
<16.9 mm (score 1)	3 (10.3)
>16.9 mm (score 2)	26 (89.7)
**Optic Canal Invasion**	
None (score 0)	1 (3.4)
Unilateral (score 1)	8 (27.6)
Bilateral (score 2)	20 (69)
**Vascular Invasion**	
None (score 0)	2 (6.9)
<180° (score 1)	9 (31)
>180° (score 2)	18 (62.1)
**Peritumoral Edema**	
Yes	2 (6.9)
No	27 (93.1)

**Table 4:** The relationship between the scoring system and the clinical signs (visual acuity and hemianopsia).

Scoring	Visual Acuity		*p* Value	Hemianopsia		*p* Value
	≥3/60	<3/60		None	Yes	
**Tumor Diameter**						
<16.9 mm	0 (0%)	3 (100%)	>0.999 *	1 (33.3%)	2 (66.7%)	>0.999 *
>16.9 mm	4 (15.4%)	22 (84.6%)		7 (35%)	13 (65%)	
**Optic Canal Invasion**						
None	1 (100%)	0 (0%)	0.076 **	0 (0%)	1 (100%)	0.657 **
Unilateral	2 (25%)	6 (75%)		3 (50%)	3 (50%)	
Bilateral	1 (5%)	19 (95%)		5 (31.3%)	11 (68.8%)	
**Vascular Invasion**						
None	2 (100%)	0 (0%)	0.622 **	0 (0%)	2 (100%)	0.371 **
<180°	0 (0%)	9 (100%)		4 (66.7%)	2 (33.3%)	
>180°	2 (11.1%)	16 (88.9%)		4 (26.7%)	11 (73.3%)	
**Onset (months)**	12 (7–24)	26 (2–96)	0.281 ***	14.3 (3–45)	24 (7–96)	0.238 ***

* Fisher Test; ** Fisher Test with cell merger; *** Mann–Whitney Test.

**Table 5:** The onset of the symptoms according to the scoring system.

Scoring	Onset (Months)	*p* Value
**Tumor Diameter**		0.660 *
<16.9 mm	22 (8–27)	
>16.9 mm	24 (2–96)	
**Optic Canal Invasion**		0.127 *
None	24	
Unilateral	12 (2–30)	
Bilateral	26.5 (4–96)	
**Vascular Invasion**		0.328 **
None	18 (12–24)	
<180°	22 (2–30)	
>180°	26.5 (4–96)	

* Mann–Whitney Test; ** Krusskal–Wallis Test. The onset of symptoms was defined as the duration of the first symptoms occurring until medical attention was sought at our institution.

**Table 6:** The relationship of the scoring system and resection rate.

Scoring	Resection Rate (%)	*p* Value
**Tumor Diameter**		0.281 *
<16.9 mm	100 (81–100)	
>16.9 mm	91.3 (10.81–100)	
**Optic Canal Invasion**		<0.001 *
None	100	
Unilateral	100 (95.61–100)	
Bilateral	77.5 (10.81–100)	
**Vascular Invasion**		0.024 *
None	100	
<180°	97.7 (10.81–100)	
>180°	82.17 (14.22–100)	

* Mann–Whitney Test.

**Table 7:** The relationship of the scoring system and clinical improvement.

Scoring	Clinical Post Surgery			*p* Value
	Improvement	Stable	Worsen	
**Tumor Diameter**				0.267 *
<16.9 mm	2 (66.7)	1 (33.3)	0 (0)	
>16.9 mm	8 (30.8)	17 (65.4)	1 (3.8)	
**Optic Canal Invasion**				>0.999 *
None	0 (0)	0 (0)	1 (100)	
Unilateral	3 (37.5)	5 (62.5)	0 (0)	
Bilateral	7 (35)	13 (65)	0 (0)	
**Vascular Invasion**				0.694 *
None	0 (0)	1 (50)	1 (50)	
<180°	3 (33.3)	6 (66.7)	0 (0)	
>180°	7 (38.9)	11 (61.1)	0 (0)	

* Fisher Test with cell merger.

**Table 8:** The histopathology profile.

Histopathological	Number (%)
Meningioma meningothelial WHO Grade 1	15 (51.7%)
Meningioma microcystic WHO Grade 1	1 (3%)
Meningioma mixed type WHO Grade 1	13 (45.3%)

## Data Availability

The datasets generated in this current study are available from the corresponding author upon reasonable request.

## Supplementary Materials

The following supporting information can be downloaded at: https://www.mdpi.com/article/10.3390/cancers15235700/s1, Table S1: Data of Patient.
