# Supplementary material for: Clinical Profile of Tuberculum Sellae Meningiomas Based on Scoring System: An Institutional Experience in Indonesia
Source: Cancers (Basel). 2023 Dec 3;15(23):5700. doi: 10.3390/cancers15235700 (PMC10705061; doi:10.3390/cancers15235700)
Supplement: Supplementary file 1 [file cancers-15-05700-s001.zip › cancers-2703663-supplementary.pdf]

Table S1: Data of Patient

Nama	Gender	Age	Hemianopsia	Visual Accu	TumorDiameter	OpticCanal	VascularInv	Edema	Approach	Clinical improvement	Histopathology	Complication
Patient 1	2	31.00	0	1	2	2	2	0	Pterional	Stable	Meningothelial + Transitional	No
Patient 2	2	41.00	2	1	2	2	1	0	COZ	Improvement	Meningothelial	No
Patient 3	2	44.00	2	0	2	1	0	0	Pterional	Stable	Meningothelial + Microcystic	No
Patient 4	2	41.00	0	1	2	2	2	0	Pterional	Stable	Meningothelial + Microcystic	No
Patient 5	2	51.00	2	1	2	2	2	0	Bifrontal	Stable	Meningothelial + Transitional	No
Patient 6	2	58.00	1	1	1	1	1	0	Pterional	Improvement	Meningothelial	No
Patient 7	2	47.00	2	1	2	2	2	0	Pterional	Stable	Meningothelial	No
Patient 8	2	43.00	2	0	2	1	2	0	Pterional	Improvement	Meningothelial	No
Patient 9	2	41.00	2	1	2	2	2	0	Pterional	Stable	Meningothelial + Fibrous	No
Patient 10	2	46.00	1	1	2	1	1	0	Pterional	Stable	Meningothelial + Fibrous + Transitional	No
Patient 11	2	52.00	2	0	2	0	0	0	Pterional	Worsen	Meningothelial + Microcystic	No
Patient 12	2	42.00	0	1	2	2	2	0	Pterional	Stable	Meningothelial + Microcystic	No
Patient 13	2	50.00	2	1	2	2	2	0	Pterional	Stable	Meningothelial + Microcystic	No
Patient 14	2	54.00	2	1	2	2	2	0	Bifrontal	Stable	Meningothelial	No
Patient 15	2	40.00	1	1	2	1	1	0	Pterional	Improvement	Meningothelial	No
Patient 16	2	38.00	1	1	2	2	2	0	Pterional	Stable	Meningothelial	No
Patient 17	2	50.00	2	1	2	2	2	0	Pterional	Improvement	Meningothelial	No
Patient 18	2	45.00	0	1	2	1	1	0	COZ	Stable	Meningothelial + Fibrous	No
Patient 19	2	58.00	1	1	2	2	2	1	Bifrontal	Stable	Meningothelial + Transitional	No
Patient 20	2	34.00	2	1	2	2	2	0	Pterional	Improvement	Meningothelial	No
Patient 21	2	52.00	2	0	2	2	2	0	Pterional	Improvement	Meningothelial	No
Patient 22	2	48.00	0	1	2	1	1	1	Pterional	Stable	Meningothelial	No
Patient 23	2	37.00	1	1	2	2	1	0	Pterional	Stable	Meningothelial	No
Patient 24	2	37.00	0	1	2	2	1	0	COZ	Stable	Micocystic	No
Patient 25	2	47.00	1	1	2	2	2	0	Pterional	Stable	Meningothelial	No
Patient 26	2	43.00	2	1	1	1	1	0	Pterional	Stable	Meningothelial + Microcystic	No
Patient 27	2	57.00	2	1	1	2	2	0	Pterional	Improvement	Meningothelial + Transitional	No
Patient 28	2	45.00	2	1	2	2	2	0	Pterional	Improvement	Meningothelial	No
Patient 29	2	40.00	1	1	2	2	2	0	Pterional	Improvement	Meningothelial	No

2 Female	0 N/A	0 >=3/60	1 <17	0 No	0 No	0 No
	1 No	1 <3/60	2 >17	1 Unilateral	1 Yes	1 Yes
	2 Yes			2 Bilateral		
